# Metabolite Profiles and Biological Activities of Different Phenotypes of Beech Mushrooms (*Hypsizygus marmoreus*)

**DOI:** 10.3390/foods13203325

**Published:** 2024-10-19

**Authors:** Sang-Wook Jeong, Hyeon Ji Yeo, Neul-I Ha, Kyung-Je Kim, Kyoung-Sun Seo, Seong Woo Jin, Young-Woo Koh, Hee Gyeong Jeong, Chang Ha Park, Seung-Bin Im

**Affiliations:** 1 Jangheung Research Institute for Mushroom Industry, Jangheung 59338, Republic of Korea; sangww@hanmail.net (S.-W.J.); gksmfdl7901@nate.com (N.-I.H.); ookingoo@empas.com (K.-J.K.); astragali@hanmail.net (K.-S.S.); jjin7684@hanmail.net (S.W.J.); yw-koh@hanmail.net (Y.-W.K.); hkjung53@naver.com (H.G.J.); 2 Department of Crop Science, Chungnam National University, 99 Daehak-ro, Yuseong-gu, Daejeon 34134, Republic of Korea; guswl7627@gmail.com; 3 Department of Smart Agriculture Management, Songho University, 210, Namsan-ro, Hoengseong-eup, Hoengseong-gun 24000, Republic of Korea

**Keywords:** *Hypsizygus marmoreus*, metabolites, phenolic compounds, antioxidant, anti-microbial, anti-inflammatory

## Abstract

Beech mushrooms (*Hypsizygus marmoreus*) are edible mushrooms commercially used in South Korea. They can be classified into white and brown according to their pigmentation. This study analyzed the metabolites and biological activities of these mushrooms. Specifically, 42 metabolites (37 volatiles, two phenolics, and three carbohydrates) were quantified in white beech mushrooms, and 47 (42 volatiles, two phenolics, and three carbohydrates) were detected in brown mushrooms. The major volatiles detected were hexanal, pentanal, 1-hexanol, and 1-pentanol. Brown mushrooms contained higher levels of hexanal (64%) than white mushrooms (35%), whereas white mushrooms had higher levels of pentanal (11%) and 1-pentanol (3%). Most volatiles were more abundant in white mushrooms than in brown mushrooms. Furthermore, brown beech mushrooms had a higher phenolic content than white mushrooms. Biological assays revealed that both types of mushroom demonstrated anti-microbial activities against bacterial and yeast pathogens and weak DPPH scavenging activity. The extracts from both mushrooms (50 μg/mL) also exhibited strong anti-inflammatory properties. Brown mushroom extracts showed higher antioxidant, anti-microbial, and anti-inflammatory properties than white mushroom extracts. This study reported that the differences in phenotype, taste, and odor were consistent with the metabolite differences between white and brown beech mushrooms, which have high nutritional and biofunctional values.

## 1. Introduction

Mushrooms, belonging to the kingdom Fungi, are fleshy fruiting bodies containing spores that can be subcategorized into edible and non-edible varieties. The former is characterized by unique tastes (umami, sweet, bitter, and palatable tastes) [1], flavors (musty, fishy, meat, nutty, woody, etc.) [2], textures (chewy or crisp), nutrient contents (protein, amino acids, carbohydrates, dietary fiber, vitamins, and minerals), and bioactive compounds (secondary metabolites) [3]. Among the edible varieties, beech mushrooms (*Hypsizygus marmoreus*) are known for their nutty flavor, luscious taste, and crisp texture [3]. They also exhibit antioxidant [4], anti-cancer [5], and anti-fungal [6] properties. Furthermore, beech mushrooms are rich in bioactive compounds, such as hypsiziprenol A9, which possesses anti-cancer effects [5]. This mushroom can be divided into brown and white beech mushrooms, exhibiting brown and white colors on their caps, respectively. Furthermore, brown beech mushrooms have a slightly more bitter taste and higher levels of crude proteins and crude ashes than white ones. The stipes of brown mushrooms contain higher levels of crude lipids than white ones [4].

Mushroom flavors are determined by the composition of their volatile (alcohols, ketones, esters, aldehydes, acids, alkanes, cyclic compounds, phenols, terpenes, and sulfur-containing compounds) and non-volatile compounds (amino acids, sugars, sugar alcohols, and nucleotides) [3,7,8,9]. Among the non-volatile amino acids, proline, glycine, alanine, valine, leucine, tyrosine, and phenylalanine are mainly responsible for imparting a bitter taste, while glutamic acid contributes to a umami taste [10]. Furthermore, several nucleotides [5′-guanosine monophosphate, 5′-inosine monophosphate, and 5′-xanthosine monophosphate] are also involved in imparting a umami taste [11], while sugars and sugar alcohols can influence sweetness [3]. In addition to non-volatiles, aliphatic C8 compounds (e.g., 1-octen-3-ol, 3-octanol, 1-octanol, 1-octen-3-one, and 3-octanone), sulfur-containing compounds (dimethyl disulfide and dimethyl trisulfide), aldehydes (hexanal, nonanal, benzaldehyde, etc.), acids (hexanoic acid, octanoic acid, nonanoic acid, etc.), alkanes (dodecane, hexadecane, etc.), alcohols (1-octen-3-ol, 3-octanol, 1-hexanol, etc.), and esters (methyl ester) are responsible for various flavors [9,12].

Phenolics are secondary metabolites biosynthesized in mushrooms. Most of the phenolic compounds present in mushroom species are phenolic acids and flavonoids [13]. Secondary metabolites from mushrooms have been reported to have potential biological activities, such as anti-inflammatory [14], anti-microbial [15], anti-diabetes [13], and antioxidant activities [16]. Taofiq et al. (2016) reported that phenolic acids, terpenoids, and polysaccharides are likely to affect the anti-inflammatory properties of mushrooms [17]. Phenolic acids are regarded as the major bioactive compounds in mushrooms. They can be categorized into two main groups: hydroxybenzoic and hydroxycinnamic acids. Mushrooms contain hydroxybenzoic acids (e.g., protocatechuic, gentisic, *p*-hydroxybenzoic, gallic, vanillic, and syringic acids) [18].

**Figure 1:**
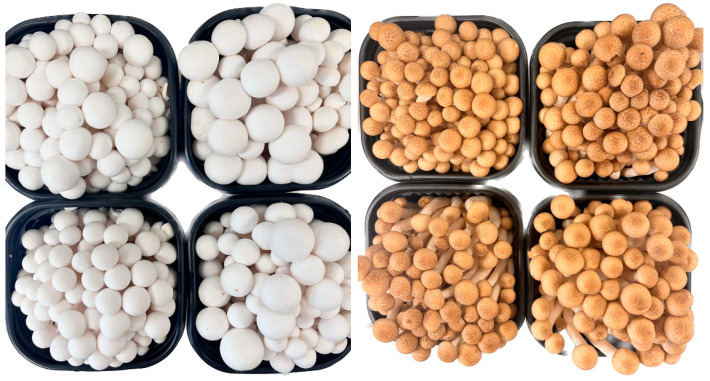
White beech mushroom (**left**) and brown beech mushroom (**right**).

Differences in the metabolites (amino acids, organic acids, sugars, sugar alcohols, and hypsiziprenols) have been reported between the caps and stipes of white and brown beech mushrooms. However, to the best of our knowledge, only a few studies have investigated the differences in metabolites, including phenolics and volatiles, and their biological activities. Thus, this present study aimed to investigate the differences in the metabolites (volatiles, phenolics, and carbohydrates) and biological activities (antioxidant, anti-microbial, and anti-inflammatory activities) between white and brown beech mushrooms (Figure 1).

## 2. Materials and Methods

### 2.1. Materials

Brown and white beech mushroom samples were obtained from Yooho Co., Ltd. (95, Haksan 1-gil, Iseo-myeon, Cheongdo-eup, Cheongdo-gun, Gyeongsangbuk-do, Republic of Korea). The mushroom samples were cultivated for 100 d using bottle technology. One bottle represented one biological replicate and four bottles for each mushroom were used in this study. The fruiting bodies of both beech mushrooms are shown in Figure 1. The 100-day fruiting bodies were harvested using liquid nitrogen, freeze-dried, and ground to a powder.

### 2.2. Determination of Volatile Compound Composition

The mushroom samples (0.5 g) were weighed and placed in a 20 mL screw-capped headspace vial. The headspace program was implemented using a ChroZen PAL RTC system. Briefly, a needle was inserted into the sample headspace and then continuously stirred (250 rpm) for 30 min at 80 °C. After extraction, the needle was removed from the vial and immediately inserted into the GC-MS sampler for desorption (at 60 °C for 10 min) and further analysis. The volatiles were studied using a triple quadrupole GC-MS system. A DB-5MS column (30 m × 0.25 mm × 0.25 µm; Agilent Technologies, Santa Clara, CA, USA) was used as the analysis column and high-purity helium was used as the carrier gas at a flow rate of 1.0 mL/min. The column temperature was initially set to 50 °C (held for 5 min) and then increased to 280 °C (held for 20 min), increasing by 3 °C/min. The MS ion source temperature and the inlet temperature were set to 230 °C and 250 °C, respectively, and the electron energy was 70 eV. The scanning range was 35–650 amu and the solvent delay time was 4 min.

### 2.3. Free Sugar Content Analysis

To determine the free sugar content, the samples (0.5 g) were extracted with distilled water (30 mL) and subjected to a water bath for 4 h at 60 °C. The water extract was separated via centrifugation at 3000 rpm for 30 min. The supernatant was filtered using a 0.45 μm syringe filter (Advantec Dismicr, Tokyo, Japan). The HPLC analysis was performed using a 1200 Series HPLC system (Agilent Technologies) with an evaporative light scattering detector (ELSD). The separation was achieved with an Agilent ZORBAX Carbohydrate column (Agilent Technologies), and the temperature was maintained at 30 °C. The chromatography parameters were as follows: a flow rate of 1.4 mL/min, an injection volume of 5 μL, and the mobile phase was acetonitrile and distilled water (75:25 *v*/*v*). The ELSD conditions were as follows: gain, 200; gas pressure, 40 psi; temperature of the drift tube, 40 °C; and nebulizer, cooling. All samples were analyzed in triplicate. The analytes were identified by comparing them with authentic high-purity standards (Sigma-Aldrich Co., Yongin, Republic of Korea)) and quantified through external standardization.

### 2.4. HPLC Analysis of Phenolic Compounds, Total Phenolic Content (TPC) Assay, and 2,2′-Diphenyl-1-picrylhydrazyl Radical (DPPH) Assay

The HPLC analysis of the phenolic compounds and the determination of the total phenolic content (TPC) were performed as described in a previous study [19,20]. Briefly, 100 mg of dried white and brown beech mushroom powder was soaked in 2 mL of MeOH (80% *v*/*v*) and sonicated for 60 min. After centrifugation at 10,000× *g* for 30 min, the supernatant was syringe-filtered through a vial. The HPLC system and conditions were the same as previously described [19]. The presence of phenolic compounds in both mushrooms was confirmed using a spike test and compared with the retention times of gallic acid and *epi*-catechin. For the TPC assay, the extracts of both mushrooms (0.1 mL), prepared as above, were added to HPLC-grade water (3.4 mL) and 2 N Folin and Ciocalteu phenol reagent (0.5 mL), followed by incubation for 5 min in the dark. After adding 2 mL of sodium carbonate (20% *w*/*v*), the mixtures were incubated in the dark for 1 d, and their absorbance was measured at 760 nm. The TPC was expressed as gallic acid equivalents (GAEs). Moreover, the DPPH scavenging activity of the white and brown beech mushroom extracts was estimated as previously described [21]. Briefly, 150 μL of 1 mM DPPH• methanol solution and 50 μL of extracts from both beech mushrooms were incubated for 10 min in the dark, followed by the absorbance measurement at 517 nm.

### 2.5. In Vitro Anti-Microbial Assay

The anti-microbial screening of the white and brown beech mushroom extracts was conducted as previously described. Five grams of dried powder from each mushroom was soaked in 30 mL of MeOH and sonicated for 60 min. After shaking for 1 d, the mixtures were centrifuged at 10,000× *g* for 30 min and then syringe-filtered, followed by evaporation. The evaporated extracts were dissolved in DMSO at a concentration of 100 mg/mL for the anti-microbial screening. Next, 0.1 mL of various microbial cultures [*Bacillus cereus* (KCTC 3624), *Proteus mirabilis* (KCTC 2510), *Vibrio parahaemolyticus* (KCTC 2471), *Staphylococcus aureus* (KCTC 3881), *Chryseobacterium gleum* (KCTC 2094), *Staphylococcus epidermidis* (KCTC3958), *Pseudomonas aeruginosa* (KCCM 11803), *Streptococcus mutans* (KCTC 3065), *P*. *aeruginosa* (0254), *P*. *aeruginosa* (0826), *P*. *aeruginosa* (1378), *P*. *aeruginosa* (1113), *P*. *aeruginosa* (1731), *P*. *aeruginosa* (1378), and *Candida albicans* (ATCC 28367)] at an OD_600_ of 1.0 were added into warm agar medium (~40 °C) and then solidified. Two sterilized discs were then placed on the solidified medium, and extracts of both white and brown beech mushrooms (5.0 mg) were added to each disc. After incubation for 1 d, the diameters of the resulting clear zones were determined.

### 2.6. Cell Culture and Treatment

Murine RAW 264.7 cells were incubated in RPMI 1640 medium with fetal bovine serum (10%), penicillin (100 U/mL), streptomycin (100 μg/mL), vitamins (1×), and L-glutamine (2 mmol/L) at 5% CO_2_ and 37 °C. For the WST-1 assay, 50 μL of WST-1 reagent was added to the cultured cells and then incubated for 30 min, followed by an absorbance measurement at 450 nm. For the anti-inflammatory assays, 50 μg/mL extracts from the white and brown beech mushrooms were used. The cells were pre-treated with these extracts for 5 h, and then 1 μg/mL of LPS was added to the cells. For the quantitative reverse transcription (qRT) PCR analysis, the cells were treated with LPS, LPS + brown beech mushroom extract, and LPS + white beech mushroom extract, including the control (untreated), and collected; then, their total RNA was isolated using a previously reported method [22]. The total RNA (1 μg) was obtained from the cells for the cDNA synthesis using a ReverTra Ace kit (Toyobo Co., Ltd., Osaka, Japan), and the resulting cDNA was diluted 10 times. Each cDNA template (5 μL) was mixed with 10 μL of BioFACT 2× Real-Time PCR Master Mix kit with SFCgreen I (BioFACT, Daejeon, Republic of Korea), 3 μL of nuclease-free water, and 1 μL of 0.5 μM of each primer set (*MCP*-1, *Il-1β*, *Il-6*, and *TNF-α*) on a PikoReal2 PCR system (Thermo Scientific, Waltham, MA, USA)

### 2.7. Statistical Analysis

SAS software version 9.4 (SAS Institute Inc., Cary, NC, USA) was used for the Duncan’s multiple range test (DMRT). The heatmap analysis, principal component analysis (PCA), and hierarchical cluster analysis with Pearson’s correlations were conducted using MetaboAnalyst 5.0 (http://www.metaboanalyst.ca/, accessed on 11 November 2023). All the values were calculated as the mean ± standard deviation of the quadruplicate experiments.

## 3. Results

### 3.1. Volatiles Composition in White and Brown Beech Mushrooms

**Figure 2:**
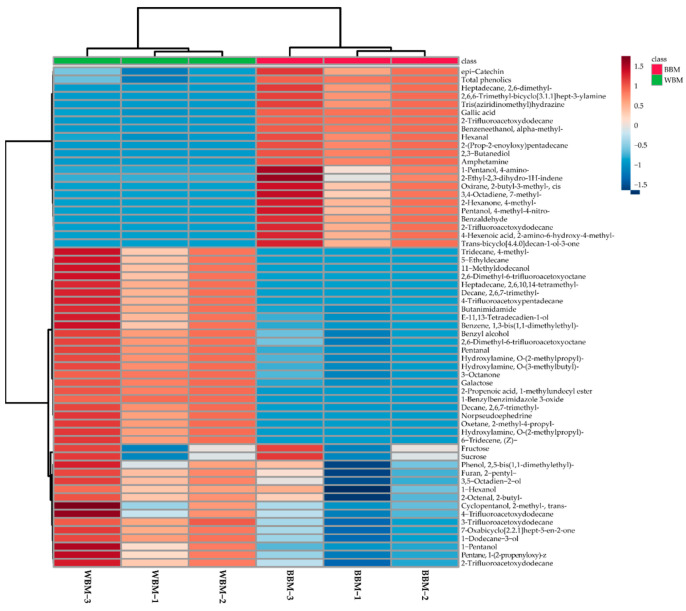
Heatmap representing the differences in the relative concentrations of the metabolites quantified in the white and brown beech mushrooms.

A total of 37 volatiles were detected in the white beech mushrooms and 42 were identified in the brown beech mushrooms (Figure 2). Of all the volatiles detected, an abundance of 24 volatiles [pentanal; hydroxylamine, O-(2-methylpropyl)-; pentane, 1-(2-propenyloxy)-; hydroxylamine, O-(3-methylbutyl)-; 1-pentanol; hexanal; cyclopentanol, 2-methyl-, trans-; E-11, 13-tetradecadueb-1-ol; 1-hexanol; 2-trifluoroacetoxydodecane; 7-oxabicyclo[2.2.1]hept-5-en-2-one; 1-dodecane-3-ol; 3-octanone; furan, 2-pentyl-; decane, 2,6,7-trimethyl-; benzyl alcohol; 3,5-octadien-2-ol; 3-trifluoroacetoxydodecane; 2,6-dimethyl-6-trifluoroacetoxyoctane; benzene, 1,3-bis(1,1-dimethylethyl)-; butanimidamide; 2-octenal, 2-butyl-; phenol, 2,5-bis(1,1-dimethylethyl)-; and 4-trifluoroacetoxypentadecane] was detected in both mushrooms. Among these, the amount of 17 volatiles (pentanal; hydroxylamine, O-(2-methylpropyl)-; pentane, 1-(2-propenyloxy)-; hydroxylamine, O-(3-methylbutyl)-; 1-pentanol; E-11, 13-tetradecadueb-1-ol; 2-trifluoroacetoxydodecane; 1-dodecane-3-ol; 3-octanone; furan, 2-pentyl-; decane, 2,6,7-trimethyl-; benzyl alcohol; 3,5-octadien-2-ol; 3-trifluoroacetoxydodecane; 2,6-dimethyl-6-trifluoroacetoxyoctane; benzene, 1,3-bis(1,1-dimethylethyl)-; and butanimidamide) was significantly higher in the white beech mushrooms than the brown beech mushrooms. However, the abundance of hexanal was significantly higher in the brown beech mushrooms than in the white beech mushrooms. Eighteen volatiles [2,3-butanediol; 2-hexanone, 4-methyl-; oxirane, 2-butyl-3-methyl-, cis; 2-trifluoroacetoxydodecane; 1-pentanol, 4-amino-; 4-hexenoic acid, 2-amino-6-hydroxy-4-methyl-; benzaldehyde; pentanol, 4-methyl-4-nitro-; 2-(prop-2-enoyloxy)pentadecane; 3,4-octadiene, 7-methyl-; amphetamine; 2,6,6-trimethyl-bicyclo[3.1.1]hept-3-ylamine; benzeneethanol, α-methyl-; tris(aziridinomethyl)hydrazine; heptadecane, 2,6-dimethyl-; 2-ethyl-2,3-dihydro-1H-indene; trans-bicyclo[4.4.0]decan-1-ol-3-one; and 2-trifluoroacetoxydodecane] were detected only in the brown beech mushrooms, while 13 volatiles [heptadecane, 2,6,10,14-tetramethyl-; hydroxylamine, O-(2-methylpropyl)-; 6-tridecene, (Z)-; 2-propenoic acid, 1-methylundecyl ester; oxetane, 2-methyl-4-propyl-; 1-benzylbenzimidazole 3-oxide; 11-methyldodecanol; 2,6-dimethyl-6-trifluoroacetoxyoctane; norpseudoephedrine; decane, 2,6,7-trimethyl-; 4-trifluoroacetoxypentadecane; tridecane, 4-methyl-; and 5-ethyldecane] were detected only in the white beech mushrooms.

### 3.2. Phenolic Compounds in White and Brown Beech Mushrooms

The HPLC analysis of the white and brown beech mushroom samples confirmed the presence of both gallic acid and *epi*-catechin (Table 1). The amount of phenolic compounds was higher in the brown beech mushrooms, at 0.102 ± 0.001 mg g ^−1^ dry weight (1.82-fold higher than in the white beech mushrooms). In particular, the levels of gallic acid and *epi*-catechin were 2.67 times and 1.18 times higher, respectively, than in the white beech mushrooms. Similarly, the TPC assay (Table 2) revealed that the TPC of the brown beech mushrooms (34.15 GAE g^−1^ dry weight) was higher than that of the white beech mushrooms (29.85 GAE g^−1^ dry weight). Moreover, the DPPH radical scavenging activity of the brown beech mushrooms was significantly higher than in the white beech mushrooms (Table 2). The DPPH activity results positively correlated with the TPC.

### 3.3. Free Sugar Content of White and Brown Beech Mushrooms

The HPLC analysis of the white and brown beech mushroom samples confirmed the presence of the free sugars fructose and galactose, which are monosaccharides, and sucrose (Table 3). The total content of these three sugars was higher in the white beech mushrooms, at 3.55 ± 0.08%, 1.91-fold higher than in the brown beech mushrooms. In particular, the level of galactose in the white beech mushrooms was 1.94 times higher than in the brown beech mushrooms (Table 3).

### 3.4. Multivariate Analysis

**Figure 3:**
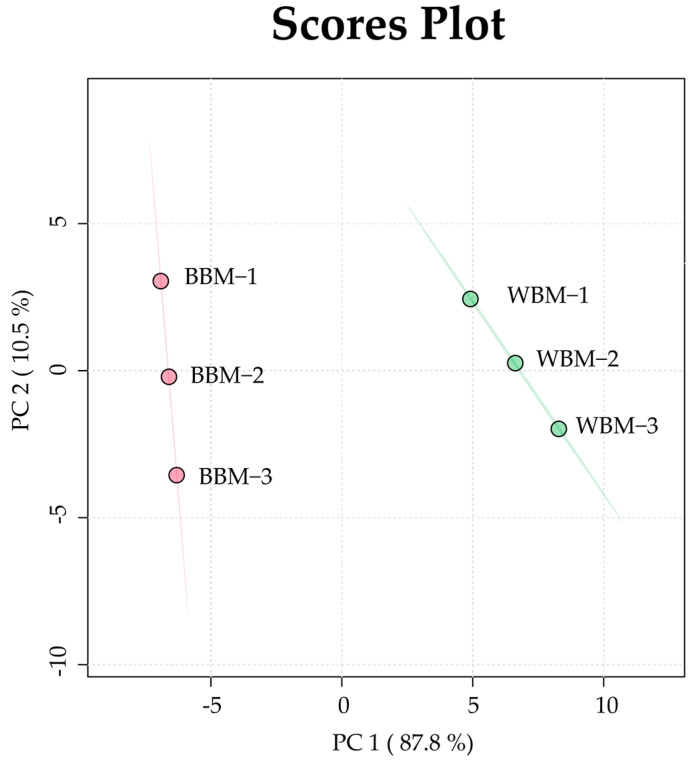
Scores plot of the principal component analysis (PCA) model obtained using metabolites from white and brown beech mushrooms.

**Figure 4:**
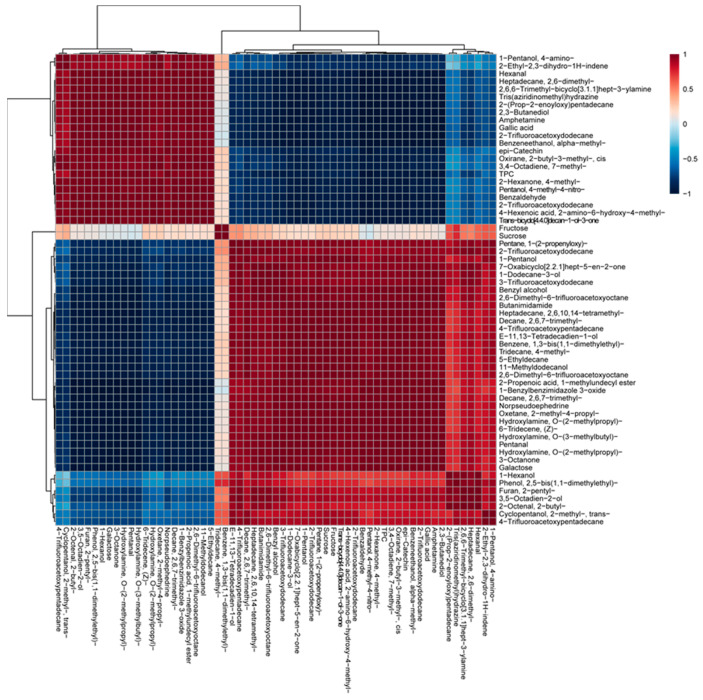
Correlation matrix of metabolites from white and brown beech mushrooms. Each square indicates the Pearson’s correlation coefficient of a pair of compounds, and the value of the correlation coefficient is represented by the intensity of the color ranging from deep blue to deep red, as indicated on the color scale.

A PCA was conducted to investigate the differences in the metabolites (volatiles, phenolics, and sugars), as seen in Figure 3 and Table S1. The first component (PC1; 87.8% of the total variance) revealed a complete separation between the white and brown beech mushrooms and was attributable to the following metabolites: decane, 2,6,7-trimethyl-; oxetane, 2-methyl-4-propyl; hydroxylamine; O-(2-methylpropyl)-, 1,6-tridecene, (Z)-, pentanal; amphetamine; gallic acid; 2,3-butanediol; benzeneethanol, alpha-methyl-; and 2-trifluoroacetoxydodecane. According to the Pearson’s correlation analysis (Figure 4), *epi*-catechin; 2-trifluoroacetoxydodecane; benzeneethanol, α-methyl-; trans-bicyclo[4.4.0]decan-1-ol-3-one; 2-trifluoroacetoxydodecane; tris(aziridinomethyl)hydrazine; heptadecane, 2,6-dimethyl-; 2-(prop-2-enoyloxy)pentadecane; 2,6,6-trimethyl-bicyclo[3.1.1]hept-3-ylamine; oxirane, 2-butyl-3-methyl-, cis; 2-hexanone, 4-methyl-; 2-ethyl-2,3-dihydro-1H-indene; 2,3-butanediol; gallic acid; 3,4-octadiene, 7-methyl-; pentanol, 4-methyl-4-nitro-; 4-hexenoic acid, 2-amino-6-hydroxy-4-methyl-; benzaldehyde; 1-pentanol, 4-amino-; hexanal; and the TPC had strong positive correlations, with *r* > 0.9 and *p* < 0.01.

### 3.5. The In Vitro Anti−Microbial Properties of White and Brown Beech Mushroom Extracts

**Figure 5:**
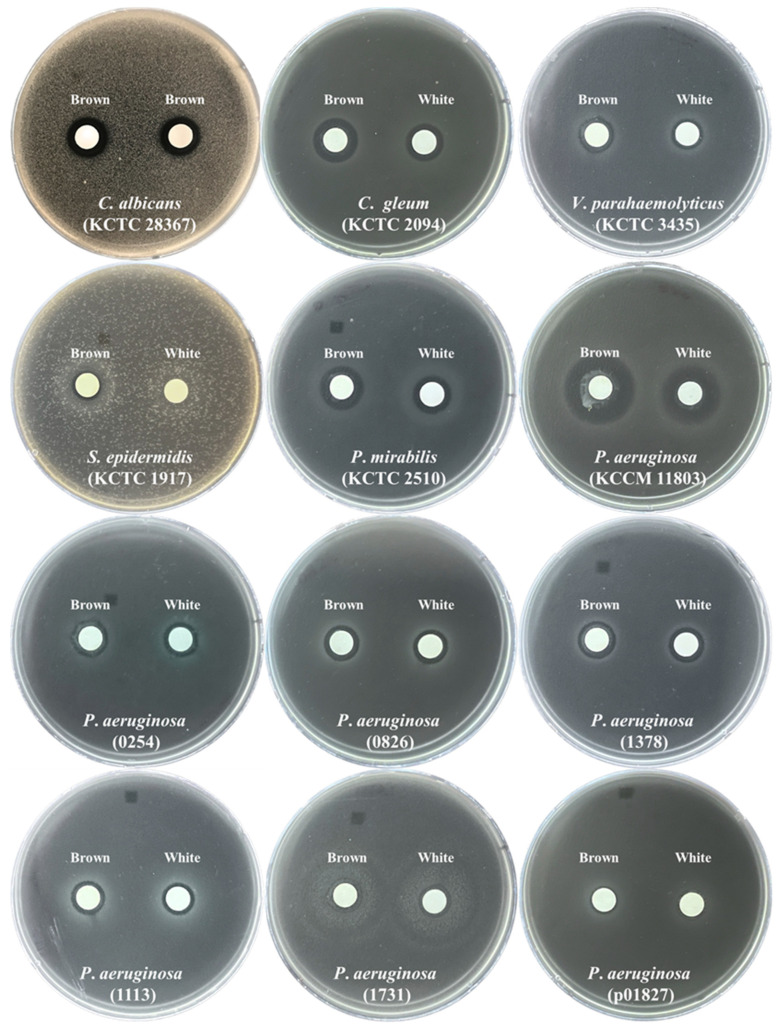
Representative images showing the anti-microbial activities of white and brown beech mushroom extracts.

The growth inhibition zones of 14 pathogenic bacteria and one pathogenic yeast were measured after the treatment with the white and brown beech mushroom extracts to evaluate the in vitro anti-microbial activities (Table 4). The anti-microbial activity of the white and brown beech mushroom extracts against five pathogens, one pathogenic yeast, and methicillin-resistant pathogens, except for *B*. *cereus* (KCTC 3624), *S*. *aureus* (KCTC 3881), and *S*. *mutans* (KCTC 3065), was confirmed (Figure 5). In particular, the brown beech mushroom extracts revealed more potent anti-microbial properties against *P*. *mirabilis* (KCTC 2510), *V*. *parahaemolyticus* (KCTC 2471), *C*. *gleum* (KCTC 2094), *S*. *epidermidis* (KCTC3958), *P*. *aeruginosa* (KCCM 11803), *C*. *albicans* (ATCC 28367), and *P*. *aeruginosa* (0254, 0826, 1378, 1113, and 1731) than the white beech mushrooms. In contrast, the white beech mushroom extract had a stronger anti-microbial effect against *P*. *aeruginosa* (0254) than the brown beech mushroom extract.

### 3.6. In Vitro Cytotoxicity of White and Brown Beech Mushroom Extracts

**Figure 6:**
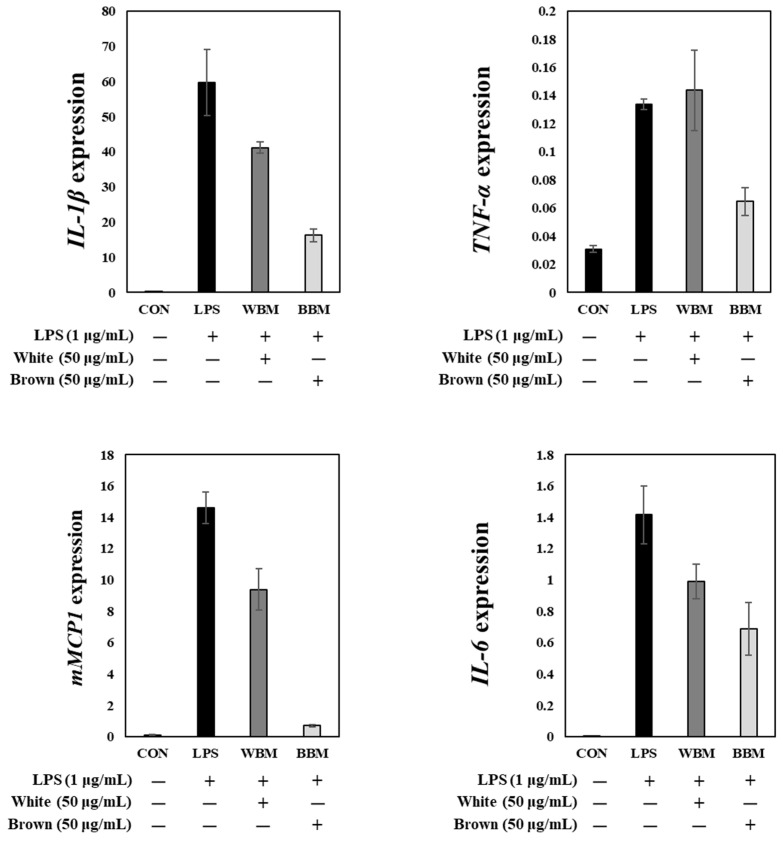
Extracts of white and brown beech mushrooms attenuated lipopolysaccharide (LPS)-induced inflammatory responses in mouse RAW264.7 macrophage cells.

The in vitro anti-inflammatory properties of the white and brown beech mushrooms were evaluated using mouse RAW264.7 macrophages (Figure 6). Based on the cytotoxicity assay results, the treatment with the mushroom extracts up to 50 μg/mL showed no significant differences in the viability of the macrophages in vitro. Thus, the expression of chemokine (*MCP-1*) and pro-inflammatory genes (*IL-1β*, *Il-6*, and *TNF-α*) was analyzed after the treatment with 50 μg/mL of the mushroom extracts. The expression levels of *TNF-α*, *IL-1β*, *Il-6*, and *MCP-1* increased upon LPS addition. In contrast, the addition of the mushroom extracts significantly reduced the expression of these genes. Specifically, the expression of *TNF-α*, *IL-1β*, *Il-6*, and *MCP-1* decreased upon treatment with the brown beech mushroom extract compared with the LPS-only treatment, while the expression of *IL-1β*, *Il-6*, and *MCP-1* decreased upon treatment with the white beech mushroom extract compared with the LPS-only treatment. Notably, the expression levels of these genes were relatively lower with the addition of the brown beech mushroom extract, indicating that the brown beech mushroom extract had a stronger anti-inflammatory effect than the white beech mushroom extract.

## 4. Discussion

Brown and white beech mushrooms have different phenotypes, flavors, and tastes. Brown beech mushrooms have a bitter taste compared with white beech mushrooms, which might be due to the higher levels of phenolic compounds in brown mushrooms, as several phenolic compounds have a bitter taste. In particular, *epi*-catechin, which was detected at higher levels in the brown mushrooms than in the white mushrooms in this study, has been reported to be a bitter-tasting compound [23]. Furthermore, bitterness can be expected to be lower in white beech mushrooms because their total sugar and galactose contents are higher than in brown beech mushrooms. The relative sweetness of galactose is 30% compared with sucrose [24]. In addition to taste differences, the phenotypic differences between white and brown beech mushrooms may also be due to the brown coloration, which results from phenolic compound production and oxidation [25]. This study showed a positive correlation between brown color and TPC (gallic acid and *epi*-catechin), consistent with a previous study reporting that brown color generation in pistachio tissue cultures positively correlated with phenolic content [26]. Beech mushrooms have a mild nutty flavor [3]. There is a difference in flavor between brown and white beech mushrooms, which may be attributed to differences in the abundance of various volatile compounds. The most abundant volatiles in both beech mushrooms were 1-hexanol, imparting grassy and green flavors [27]; 1-pentanol, imparting balsamic and fruity flavors [27]; hexanal, imparting fatty, oily, and grassy flavors [28]; and pentanal, imparting pungent, almond, and malt flavors [29]. Brown beech mushrooms have a higher hexanal content, whereas 1-pentanol and pentanal were more abundant in white beech mushrooms. Therefore, these results suggest that brown beech mushrooms are characterized by a grassy flavor due to their high abundance of hexanal, while white beech mushrooms have a more nutty flavor, as evidenced by their higher levels of pentanal.

According to the PCA analysis, white beech mushrooms were completely separated from brown beech mushrooms. This finding implies that there are metabolite differences between them. Among the metabolites, volatiles belonging to alkanes, amides, alcohols, and galactose were the most abundant in white beech mushrooms. In contrast, phenolic compounds (gallic acid and *epi*-catechin) were more abundant in brown beech mushrooms. These results show that metabolite content, phenotype, flavor, and taste differences exist between brown and white beech mushrooms.

Our results also showed that the white and brown beech mushroom extracts showed strong anti-microbial properties against *P*. *mirabilis* (KCTC 2510), *V*. *parahaemolyticus* (KCTC 2471), *C*. *gleum* (KCTC 2094), *S*. *epidermidis* (KCTC3958), *P*. *aeruginosa* (KCCM 11803), methicillin-resistant *P*. *aeruginosa* (0254, 0826, 1378, 1113, 1731, and 1378), and *C*. *albicans* (ATCC 28367). These results agree with those of previous studies reporting the anti-microbial activities of mushrooms belonging to the genus *Hypsizygus*. For instance, the extract of *H*. *marmoreus* mycelia inhibited the growth of *P*. *aeruginosa* and *C*. *albicans* [30]; and the extracts of *H*. *tessellatus* inhibited the growth of *E*. *coli*, *S*. *marcescens*, *S*. *aureus*, and *B*. *subtilis* [31]. Furthermore, in this study, brown beech mushrooms showed higher anti-microbial activity than white beech mushrooms, which may be attributed to the higher levels of phenolic compounds (gallic acid and *epi*-catechin) in brown beech mushrooms than in white ones. Phenolic compounds have been reported to exert anti-microbial effects. Gallic acid and *epi*-catechin are prominent phenolic compounds with anti-microbial activity. In particular, gallic acid directly inhibits the growth of *E*. *coli*, *P*. *aeruginosa*, *S*. *aureus*, *Listeria monocytogenes* [32], multidrug-resistant *E*. *coli* [33], *Klebsiella pneumoniae*, and *Shigella flexneri* [34], while enhancing the anti-microbial effectiveness of several antibiotics [35]. *epi*-Catechin has been shown to inhibit the growth of *B*. *subtilis*, *B*. *cereus*, *S*. *aureus*, *L*. *monocytogenes*, *E*. *coli*, *Salmonella typhimurium*, *P*. *aeruginosa*, and *V*. *parahaemolyticus*. Furthermore, brown beech mushrooms contain higher levels of hexanal, a naturally occurring anti-microbial volatile compound [36], than white beech mushrooms.

In addition to their anti-microbial effects, the extracts of brown and white beech mushrooms also exhibit anti-inflammatory effects, consistent with previous studies reporting the anti-inflammatory activities of mushrooms belonging to the genus *Hypsizygus*. For example, Chien et al. (2016) reported that the addition of *H*. *marmoreus* extracts suppressed the production of IL-6, TNF-α, and IL-1β in RAW 264.7 cells [37]. Moreover, Al-Faqeeh et al. (2023) demonstrated that *H*. *ulmarius* extracts inhibited the activities of lipoxygenase (LOX), cyclooxygenase (COX), and myeloperoxidase (MPO), which are enzymes mediating inflammation [38]. Moreover, brown beech mushroom extracts exhibited stronger anti-inflammatory effects than white beech mushroom extracts, as evidenced by the reduction in *TNF-α*, *IL-1β*, *Il-6*, and *MCP-1* expression. This may be attributed to the high levels of phenolics in brown beech mushrooms, which have been reported to have anti-inflammatory effects [39]. *epi*-Catechin and gallic acid are phenolic compounds with anti-inflammatory properties. *epi*-Catechin has been reported to inhibit TNF-α-induced inflammatory responses by inhibiting *TNF-α*, *Il-6*, and *MCP-1* expression [40]. Similarly, gallic acid has been reported to suppress *TNF-α*, *IL-1β*, and *Il-6* expression [41].

Brown beech mushrooms also showed higher antioxidant activity than white beech mushrooms, as evidenced by the results of the DPPH assay. This may be attributed to the high content of phenolics in brown beech mushrooms, which are strong antioxidants [42]. Collectively, our results suggest that brown beech mushrooms exhibit greater anti-microbial, antioxidant, and anti-inflammatory effects than white beech mushrooms, owing to their higher levels of phenolic compounds. However, there is often no strong correlation between in vitro and in vivo results, which limits clinical potential. Therefore, further studies are required to evaluate the in vivo antioxidant, anti-microbial, and anti-inflammatory activities of white and brown beech mushrooms.

## 5. Conclusions

In conclusion, the results of our metabolite analyses and bioassays (antioxidant, anti-microbial, and anti-inflammatory) on brown and white beech mushrooms showed phenotypic, odor, taste, and metabolite differences between the two beech mushrooms. The bitter taste of brown beech mushrooms can be explained by the high levels of *epi*-catechin, a bitter-tasting compound, and the brown coloration in brown beech mushrooms can be explained by their high levels of phenolic compounds. Furthermore, this study suggests that brown beech mushrooms are sweeter than white beech mushrooms because they contain higher levels of galactose. In addition to taste, our results also suggested odor differences between the two beech mushrooms, with hexanal (fatty, oily, and grassy odors) being more abundant in brown beech mushrooms and pentanal (pungent, almond, and malt odors) and 1-pentanol (balsamic and fruity odors) being more abundant in white beech mushrooms. Furthermore, metabolite differences were observed between the mushrooms, with higher levels of most volatiles and sugars in the white beech mushrooms and higher levels of phenolic compounds in the brown beech mushrooms. The results of the biological activity assays suggest that both beech mushrooms showed effective antioxidant, anti-microbial, and anti-inflammatory properties, with brown beech mushrooms showing higher activity than white beech mushrooms. This might be due to the higher levels of phenolic compounds in the brown beech mushrooms, which possess antioxidant, anti-microbial, and anti-inflammatory effects, as well as hexanal, which has anti-microbial effects. Collectively, the results of this study suggest that brown and white beech mushrooms could be good food sources because of their high nutritional and biofunctional values.

## Figures and Tables

**Table 1:** Phenolic contents in white and brown beech mushrooms (mg g ^−1^ dry weight).

	Gallic Acid (mg g ^−1^ Dry Weight)	*epi*-Catechin (mg g ^−1^ Dry Weight)	Sum (mg g ^−1^ Dry Weight)
White beech mushroom	0.024 ± 0.001	0.033 ± 0.001	0.056 ± 0.002
Brown beech mushroom	0.064 ± 0.001 ***	0.039 ± 0.001 **	0.102 ± 0.001 ***

Asterisks indicate significant differences between white and brown beech mushrooms, as determined using Student’s *t*-test (** *p* < 0.01, and *** *p* < 0.001).

**Table 2:** Total phenolic content (TPC) and DPPH radical scavenging activity of white and brown beech mushrooms.

	TPC [mg Gallic Acid Equivalent (GAE)/g Dry Weight]	DPPH Radical Scavenging Activity (%)
White beech mushroom	29.85 ± 0.66	24.63 ± 2.00
Brown beech mushroom	34.15 ± 0.15 ***	42.13 ± 3.61 **

Asterisks indicate significant differences between white and brown beech mushrooms, as determined using Student’s *t*-test (** *p* < 0.01, and *** *p* < 0.001).

**Table 3:** Free sugar contents in white and brown beech mushrooms.

	Representative Sugars (%)			
	Fructose	Galactose	Sucrose	Total
White beech mushroom	0.03 ± 0.01	2.62 ± 0.08 ***	0.01 ± 0.01	3.55 ± 0.08 ***
Brown beech mushroom	0.03 ± 0.01	1.35 ± 0.07	0.01 ± 0.01	1.86 ± 0.08

Asterisks indicate significant differences between white and brown beech mushrooms, as determined using Student’s *t*-test (*** *p* < 0.001).

**Table 4:** Anti-microbial activity of the white and brown beech mushroom extracts.

Group	Bacterial Strains	Zone of Inhibition (mm)	
		Brown	White
Pathogens	*Bacillus cereus* (KCTC 3624)	- ^1^	-
	*Proteus mirabilis* (KCTC 2510)	13.8 ± 0.26 ***^2^	12.06 ± 0.17
	*Vibrio parahaemolyticus* (KCTC 2471)	14.59 ± 0.14 ***	11.93 ± 0.03
	*Staphylococcus aureus* (KCTC 3881)	-	-
	*Chryseobacterium gleum* (KCTC 2094)	14.59 ± 0.14 **	11.93 ± 0.03
	*Staphylococcus epidermidis* (KCTC3958)	9.76 ± 0.14	ND
	*Pseudomonas aeruginosa* (KCCM 11803)	23.20 ± 0.23 ***	18.71 ± 0.46
	*Streptococcus mutans* (KCTC 3065)	-	-
Pathogenic yeast	*Candida albicans* (ATCC 28367)	13.24 ± 0.15 ***	12.25 ± 0.09
Multidrug-resistant pathogens (MRPAs)	*Pseudomonas aeruginosa* (0254)	11.94 ± 0.21 **	10.80 ± 0.10
	*Pseudomonas aeruginosa* (0826)	11.68 ± 0.23 *	11.08 ± 0.08
	*Pseudomonas aeruginosa* (1378)	12.27 ± 0.19 **	11.36 ± 0.16
	*Pseudomonas aeruginosa* (1113)	10.79 ± 0.09 **	10.27 ± 0.11
	*Pseudomonas aeruginosa* (1731)	11.87 ± 0.07 ***	10.69 ± 0.14
	*Pseudomonas aeruginosa* (1378)	9.20 ± 0.07	10.15 ± 0.03 **

^1^ denotes negative; ^2^ asterisks indicate significant differences between white and brown beech mushrooms, as determined using Student’s *t*-test (* *p* < 0.05, ** *p* < 0.01, and *** *p* < 0.001).

## Data Availability

The original contributions presented in the study are included in the article/Supplementary Materials, further inquiries can be directed to the corresponding authors.

## Supplementary Materials

The following supporting information can be downloaded at: https://www.mdpi.com/article/10.3390/foods13203325/s1, Table S1: PCA scores corresponding to loading plot.
